# Investigations on the impact of nickel doping on thermoelectric properties in n-type Bi_1.8_Sb_0.2_Te_3_ alloy

**DOI:** 10.1039/d5ra05504k

**Published:** 2025-09-18

**Authors:** Poojitha G., Gurukrishna K., Srihari N. V., Poornesh P., Ashok Rao, Y. K. Kuo, Dhanya Sunil

**Affiliations:** a Department of Physics, Manipal Institute of Technology, Manipal Academy of Higher Education Manipal 576104 India a.rao@manipal.edu; b Department of Materials Science and Engineering, Indian Institute of Technology Kanpur Kanpur Uttar Pradesh 208016 India; c Department of Physics, National Dong-Hwa University Hualien 974 Taiwan ykkuo@gms.ndhu.edu.tw; d Department of Chemistry, Manipal Institute of Technology, Manipal Academy of Higher Education Manipal 576104 India

## Abstract

The incorporation of transition metals into alloys allows for the modification of carrier concentration and the optimization of electrical transport properties. This study investigates the impact of Ni on the thermoelectric properties of the Bi_1.8_Sb_0.2_Te_3_ alloy. Samples of Bi_1.8_Sb_0.2_Te_3_/*x*%Ni with varying Ni concentrations (*x* = 0, 1, 2, 3, 4, and 5) were synthesized using the solid-state reaction method, resulting in homogeneous, dense compounds characterized by a rhombohedral crystal structure and granular morphology. The electrical resistivity exhibits a gradual increase with increase in Ni concentration, attributed to enhanced electron scattering that diminishes carrier mobility. The negative Seebeck coefficients suggest that electron-dominant charge carriers predominantly influence the thermoelectric transport of the Bi_1.8_Sb_0.2_Te_3_ system. The maximum Seebeck coefficient recorded is approximately 185 μV K^−1^ for the Bi_1.8_Sb_0.2_Te_3_/3%Ni sample. The lattice thermal conductivity significantly contributes to the overall thermal conductivity, which increases with the addition of Ni due to the reduction in point defect scattering resulting from the Ni incorporation. The pristine sample demonstrates the lowest thermal conductivity of 19 mW cm^−1^ K^−1^ at 350 K. The highest power factor is approximately 1.56 mW mK^−2^ at 350 K, which is nearly identical for samples with compositions *x* = 0 and *x* = 1% Ni. The highest figure of merit (*ZT*) achieved is approximately 0.27 for the pristine sample at 350 K.

## Abstract 2

Summary of the methodology and interdependency results of thermoelectric properties of Bi_1–8_Sb_0.2_Te_3_/*x*% Ni (*x* = 0–5) compounds.

## Graphical abstract

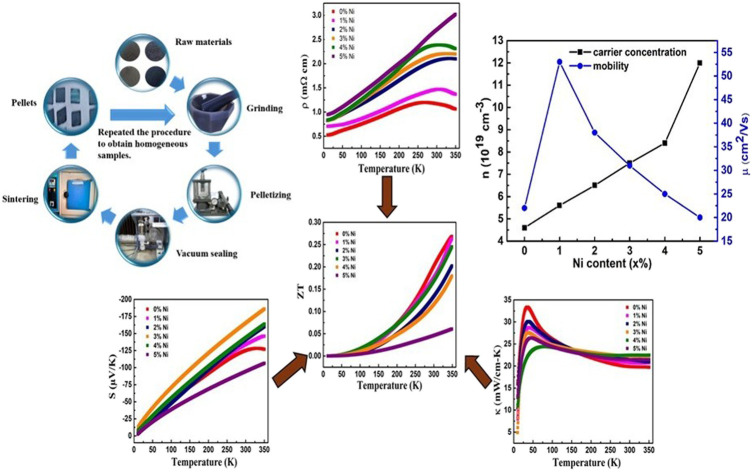

## 1. Introduction

In recent years, interest in clean energy technologies has significantly increased due to the global energy crisis. Among these innovations, thermoelectric (TE) technology stands out for its ability to directly convert waste heat into electricity.^1^ This waste heat, generated by industries, automobile exhausts, and various other sources, can be recovered and converted into useful electrical energy through TE devices made from specialized thermoelectric materials. These devices are noise-free, highly reliable and lightweight and produce no greenhouse gas emissions and have no mechanical moving parts.^2^ The efficiency of TE materials is determined by the figure of merit, represented as *ZT* = *S*^2^*σT*/*κ*_tot_, where *S*, *σ*, *T*, and *κ*_tot_ are Seebeck coefficient, electrical conductivity, absolute temperature, and total thermal conductivity, respectively.^3^ Materials with high *ZT* are suitable for practical applications of thermoelectric devices; however, achieving high *ZT* is challenging due to the strong interrelation among *S*, *σ*, and *κ*_tot_.^4^

Bismuth chalcogenides and their alloys, specifically Sb_2_Te_3_ (p-type) and Bi_2_Te_3_ (n-type), exhibit high anisotropic properties near room temperature, making them commercially used in thermoelectric devices.^5^ Additionally, solid solutions of Bi–Sb–Te (BST) alloys have effectively enhanced TE properties due to the alloying effect.^6^ While BST alloys primarily display p-type behavior, they also exhibit both n-type and p-type conductivity in Bi_2−*x*_Sb_*x*_Te_3_, where *x* ≤ 0.5 and *x* ≥ 0.5, respectively.^7,8^ The n-type BST alloys are promising candidates for thermoelectric applications and could potentially replace Bi_2_Te_3−*x*_Se_*x*_ materials. This is due to the fact that n-type Bi_2−*x*_Sb_*x*_Te_3_ materials exhibit larger mass fluctuations, have lower electronegativity, and possess a host atom with an atomic radius similar to that of the dopant compared to Bi_2_Te_3−*x*_Se_*x*_ materials. Consequently, the point defects created can lead to the scattering of short-wavelength phonons, which affects carrier transport and optimizes electrical properties.^9^

It is convenient for TE devices to have the same base materials for both legs to control the mismatch in thermal expansion coefficients and to enhance mechanical properties, ultimately improving the reliability of the device.^9^ Zhao *et al.* optimized the carrier concentration of n-type BST alloys by stoichiometrically tuning the ratio of Bi to Sb, and they obtained a carrier concentration of approximately 10^19^/cm^3^ and a power factor of around 3 μW cm^−1^ K^−2^ for Bi_1.5_Sb_0.5_Te_3_ and Bi_1.8_Sb_0.2_Te_3_.^8^ Gerovac *et al.* synthesized Bi_1.4_Sb_0.6_Te_3_ by combining melting and powder metallurgy techniques. After annealing, the material exhibited good TE efficiency with a *ZT* value of 0.5.^10^ Im *et al.* examined the TE properties of hot-pressed n-type Bi_1.8_Sb_0.2_Te_3_ single crystals, which achieved a maximum *ZT* of 0.7 and a thermal conductivity of less than 1 W m^−1^ K^−1^ in the temperature range of 250 to 300 K.^11^ However, the thermoelectric properties of these n-type materials can be enhanced by optimizing the carrier concentration and increasing phonon scattering to lower thermal conductivity through various strategies.

Elemental doping is an effective strategy for enhancing TE performance by optimizing carrier concentration through intrinsic excitation. Introducing foreign elements or compounds into the host matrix results in changes in charge mobility due to charge scattering.^12^ This process suppresses mobile charges and leads to mixed conduction. Additionally, lattice thermal conductivity can be reduced through various phonon scattering mechanisms, which are primarily influenced by the type of dopant introduced into the alloy or compound matrix. A dopant with a smaller radius occupies the interstitial sites, leading to interstitial point defect scattering of phonons. This new structure decreases the relaxation time and mean free path of the phonons, thereby increasing the probability of phonon collisions.^13^ Wei *et al.* studied the temperature-dependent properties of diamagnetic (Pb) and ferromagnetic (Fe/Co) elements-doped BST alloys. They reported that ferromagnetic elements with higher thermal activation energies do not easily enter the lattice of the host matrix. However, doping with Fe/Co is highly effective in regulating carrier concentration. This process introduces an additional carrier scattering mechanism that reduces thermal conductivity, resulting in a high *ZT* of 1.1 for 1.8% Fe doping.^14^ Mi *et al.* investigated the impact of different metals used as electrodes and barrier layers on the TE properties of Bi_0.5_Sb_1.5_Te_3_ alloy by doping it with 0.1% of Sn, Ni, Ag, and Cu. This study provides insights into the interfacial diffusion of metals into the TE legs of the module, highlighting the appropriate use of these metals as electrodes and barrier layers in TE devices at specific temperatures.^15^ Recent studies on Ni-doped p-type Bi–Sb–Te materials offer valuable insights into the role of Ni in modifying thermoelectric properties. Bano *et al.* reported that the formation of NiTe_2_ nano-inclusions creates carrier filtering channels, resulting in enhanced electrical transport properties.^16^ Hu *et al.* investigated Ni-doped Bi_0.5_Sb_1.5_Te_3_ and found that the formation of NiTe secondary phases at higher Ni concentrations led to a decrease in the figure of merit (*ZT*).^17^ Another study demonstrated that substitution of Ni into the Sb site in Bi_0.5_Sb_1.5_Te_3_ suppresses intrinsic excitation, and mass fluctuation between Ni and Sb enhances phonon scattering, thereby reducing thermal conductivity and increasing the overall *ZT* of the Ni-doped samples.^18^ However, Ni inclusion on n-type Bi–Sb–Te materials has been largely unexplored, particularly its effects at low temperatures. Therefore, the present work focuses on Ni composite of n-type Bi–Sb–Te system and investigate the impact on its transport properties in the low-to-mid temperature range. The n-type Bi_1.8_Sb_0.2_Te_3_/*x*%Ni composites prepared using the solid-state reaction method. We also conducted a detailed investigation of their structural and thermoelectric properties.

## 2. Methodology

Polycrystalline samples of Bi_1.8_Sb_0.2_Te_3_ were prepared using the solid-state reaction method, followed by a sintering process. High-purity elemental powders of Bi (99.5%, Alfa Aesar), Sb (99.999%, Good Fellow), and Te (99.999%, Alfa Aesar) were combined in stoichiometric ratios and ground in an agate mortar for 3 hours. The resulting powder was compacted into pellets using a hydraulic press that applied a pressure of 5 tons. These pellets were then sealed in quartz tubes under a high vacuum of 10^−6^ torr and sintered at 703 K for 24 hours in a muffle furnace. After sintering, the pellets were allowed to cool naturally to room temperature. The sintered pellets were ground into fine powders, and Ni (99.9%, Alfa Aesar) was incorporated in varying amounts (0 ≤ *x* ≤ 5) to create Bi_1.8_Sb_0.2_Te_3_/*x*%Ni composites. The composite powders were subsequently heated at 523 K to complete the synthesis process.

Further, the structural and thermoelectric properties of the prepared composites were examined. X-ray diffraction (XRD) data were collected at room temperature using a Rigaku Miniflex 600. Scanning electron microscopy (SEM) images were obtained with an EVO MA 18 instrument to examine the morphology of the samples. Elemental mapping was performed using energy dispersive spectroscopy (EDS) with the Oxford system. X-ray photoelectron spectroscopy was carried out using Thermo Scientific XPS system employing Al Kα radiation, with survey scans from 0 to 1300 eV at a pass energy of 200 eV. The thermoelectric properties, including temperature-dependent electrical resistivity, were measured using a four-probe method over a temperature range of 10 to 350 K. The Seebeck coefficient and thermal conductivity were measured simultaneously from 10 to 350 K using a direct heat pulse technique. Hall effect measurements were conducted at room temperature with a magnetic field of 0.5 T, utilizing the Ecopia HMS-5500 system.

## 3. Results and discussion

### 3.1 X-ray diffraction studies

**Fig. 1:**
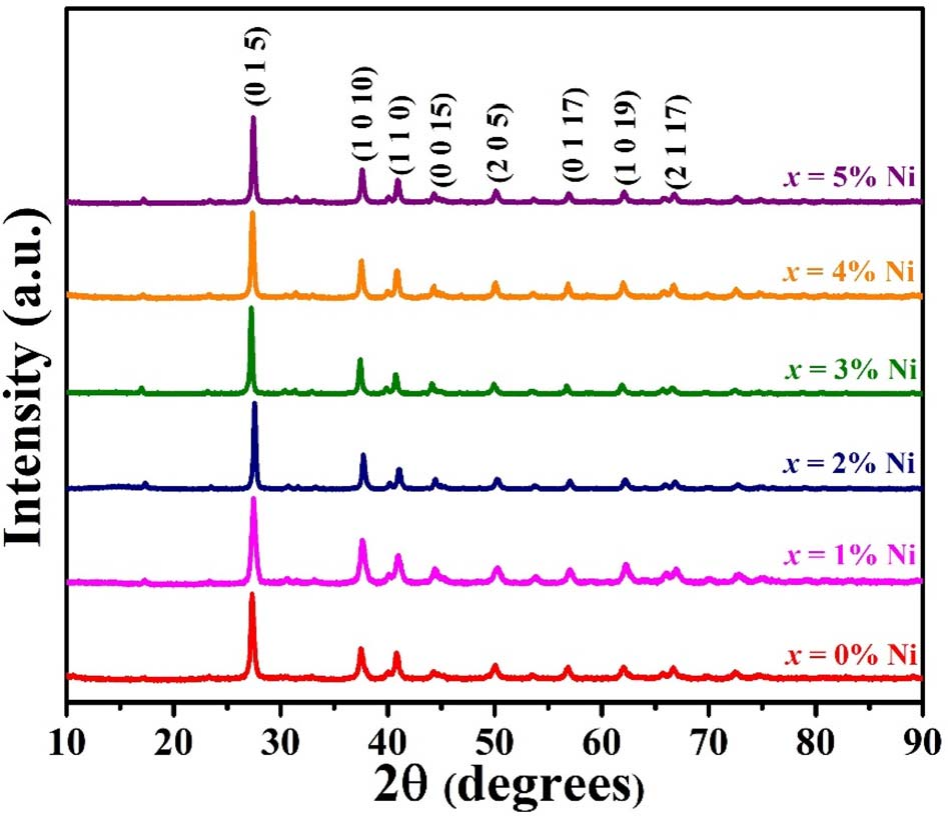
XRD plots of Bi_1.8_Sb_0.2_Te_3_/*x*%Ni (*x* = 0, 1, 2, 3, 4, and 5) samples.

**Fig. 2:**
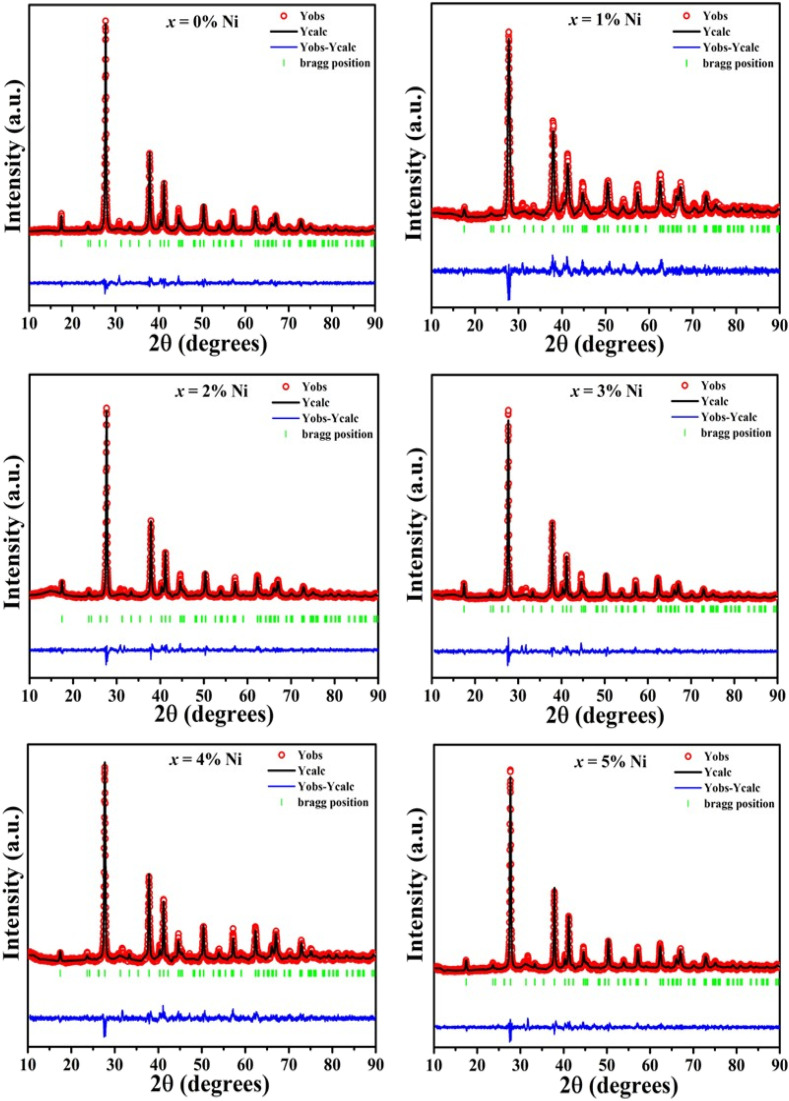
Rietveld refinement of the Bi_1.8_Sb_0.2_Te_3_/*x*%Ni (*x* = 0, 1, 2, 3, 4, and 5) samples.

**Fig. 3:**
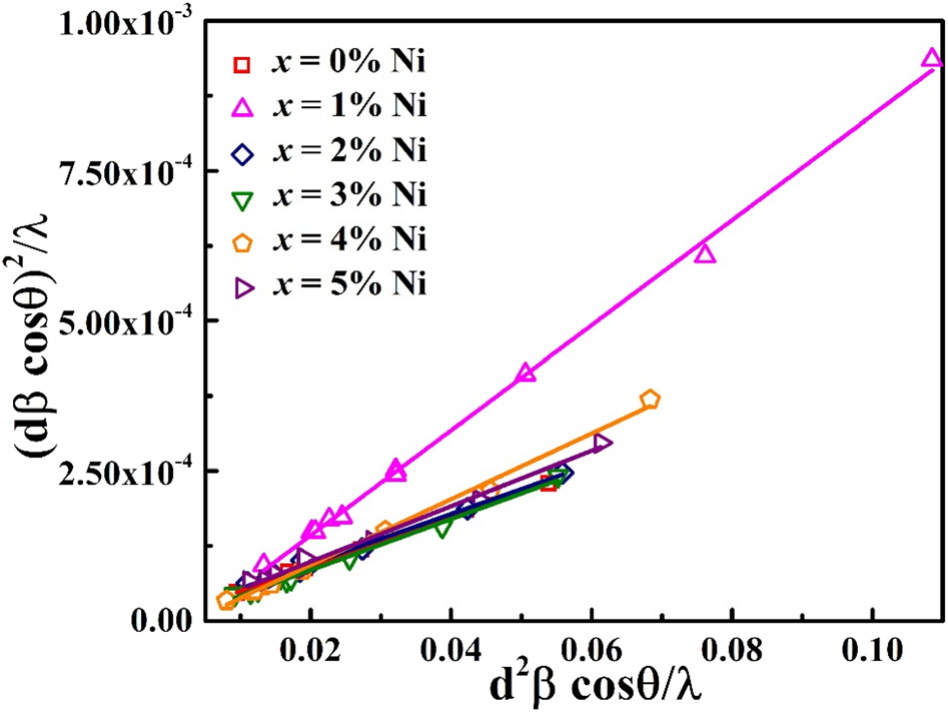
Size strain plot of the Bi_1.8_Sb_0.2_Te_3_/*x*%Ni (*x* = 0, 1, 2, 3, 4, and 5) samples.

The XRD profile of the prepared polycrystalline Bi_1.8_Sb_0.2_Te_3_/*x*%Ni (*x* = 0, 1, 2, 3, 4, and 5) composites, as shown in Fig. 1, matches well with the JCPDS card #491713, which corresponds to a rhombohedral crystal structure with *R*3̄*m* space group.^19^ For the quantitative phase analysis of the XRD profile, Rietveld refinement was performed using the FullProf suite software, as displayed in Fig. 2. During the refinement process, the Pseudo-Voigt function was used as the peak profile function, and linear interpolation was employed to describe the background variation. The observed data aligned closely with the calculated data, yielding a goodness-of-fit (GOF) index of approximately 1.3 to 1.4. The refinement parameters for all samples are listed in Table 1. It was noted that the lattice constants and cell volume decreased by 1% Ni addition, which might be attributed to the partial replacement of the Bi/Sb atoms (ionic radius of Bi^3+^ and Sb^3+^ are 1.03 Å and 0.76 Å) by smaller Ni atoms (0.69 Å). The lattice parameters remain nearly constant for *x* ≥ 2% Ni, indicating that Ni^2+^ ions occupy the interstitial site, approaching the solubility limit of Ni in these Bi_1.8_Sb_0.2_Te_3_ composites.^14,20^ The crystallite size of the composites was calculated using both the Scherrer method and the size-strain plot with the following equations,^18^1
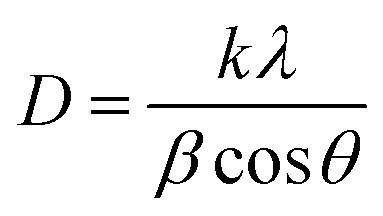
2
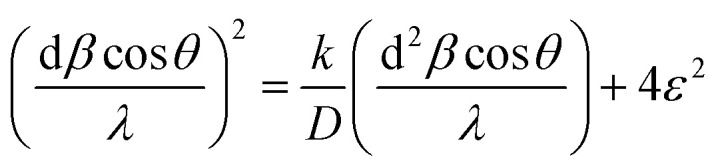
where *k* = 0.9 is the shape factor, *D* represents the crystallite size, *λ* = 1.5406 Å defines the wavelength of Cu Kα radiation (X-ray source), *β* signifies full width at half maxima of diffraction peak, and *ε* is the lattice strain. The crystallite size calculated using the size strain plot, as presented in Table 2 and depicted in Fig. 3, is more reliable than that obtained by the Scherrer method (see Table 2).^21^ The Scherrer formula has a greater margin of error because it does not account for microstrain, which contributes to peak broadening. This peak broadening is related to the increased level of polycrystallinity.^22^ The crystallite size of the Bi_1.8_Sb_0.2_Te_3_/1%Ni sample is the smallest among the samples studied, attributed to the increase in full width at half maximum (FWHM).

**Table 1:** Rietveld refinement parameters of Bi_1.8_Sb_0.2_Te_3_/*x*%Ni (*x* = 0, 1, 2, 3, 4, and 5) samples

Bi_1.8_Sb_0.2_Te_3_/*x*%Ni	*x* = 0	*x* = 1	*x* = 2	*x* = 3	*x* = 4	*x* = 5
System	Rhombohedral	Rhombohedral	Rhombohedral	Rhombohedral	Rhombohedral	Rhombohedral
Space group	*R*3̄*m*	*R*3̄*m*	*R*3̄*m*	*R*3̄*m*	*R*3̄*m*	*R*3̄*m*
*a* = *b* (Å)	4.3814(2)	4.3697(4)	4.3828(3)	4.3805(3)	4.3804(3)	4.3807(2)
*c* (Å)	30.464(2)	30.382(4)	30.469(2)	30.458(2)	30.451(2)	30.463(2)
*α* = *β* (°)	90	90	90	90	90	90
*γ* (°)	120	120	120	120	120	120
Cell volume (Å^3^)	506.47(5)	502.40(1)	506.89(6)	506.18(6)	506.03(6)	506.29(5)
*u*	0.126	0.300	0.412	0.329	0.253	0.547
*v*	−0.015	−0.374	−0.238	−0.283	−0.313	−0.387
*w*	0.043	0.278	0.065	0.050	0.123	0.104
*R*_p_	7.89	9.61	8.19	8.72	7.66	6.60
*R*_wp_	10.3	12.1	10.5	11.3	9.68	8.61
*R*_exp_	7.75	8.64	7.49	8.04	6.86	6.09
*χ* ^2^	1.75	1.91	1.97	1.97	1.99	2.00
GOF	1.3	1.4	1.4	1.4	1.4	1.4

**Table 2:** Crystallite size and microstrain of Bi_1.8_Sb_0.2_Te_3_/*x*%Ni (*x* = 0, 1, 2, 3, 4, and 5) samples

Bi_1.8_Sb_0.2_Te_3_/*x*%Ni	*x* = 0	*x* = 1	*x* = 2	*x* = 3	*x* = 4	*x* = 5
Crystallite size (Scherrer) in nm ± 3 nm	30.39	18.27	29.02	32.89	30.89	28.11
Crystallite size (size-strain plot) in nm ± 1 nm	33.41	15.82	33.41	32.54	25.25	29.94
Strain	0.0013	0.0028	0.0017	0.0005	0.0020	0.0011

### 3.2 SEM and EDS analysis

**Fig. 4:**
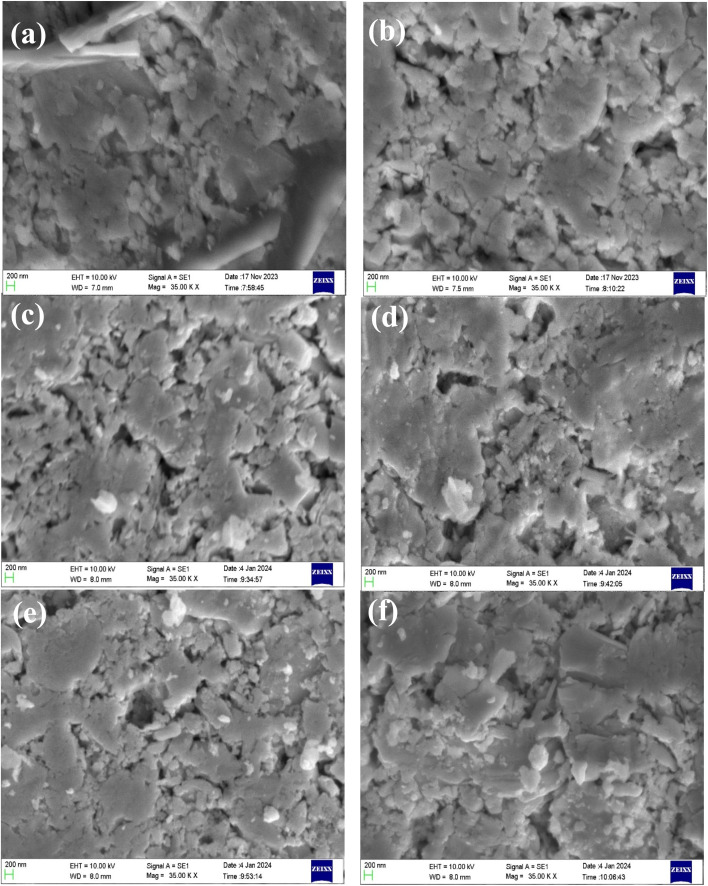
Typical SEM images of Bi_1.8_Sb_0.2_Te_3_/*x*%Ni (a) *x =* 0, (b) *x =* 1, (c) *x* = 2, (d) *x* = 3, (e) *x* = 4 and (f) *x* = 5 samples.

**Fig. 5:**
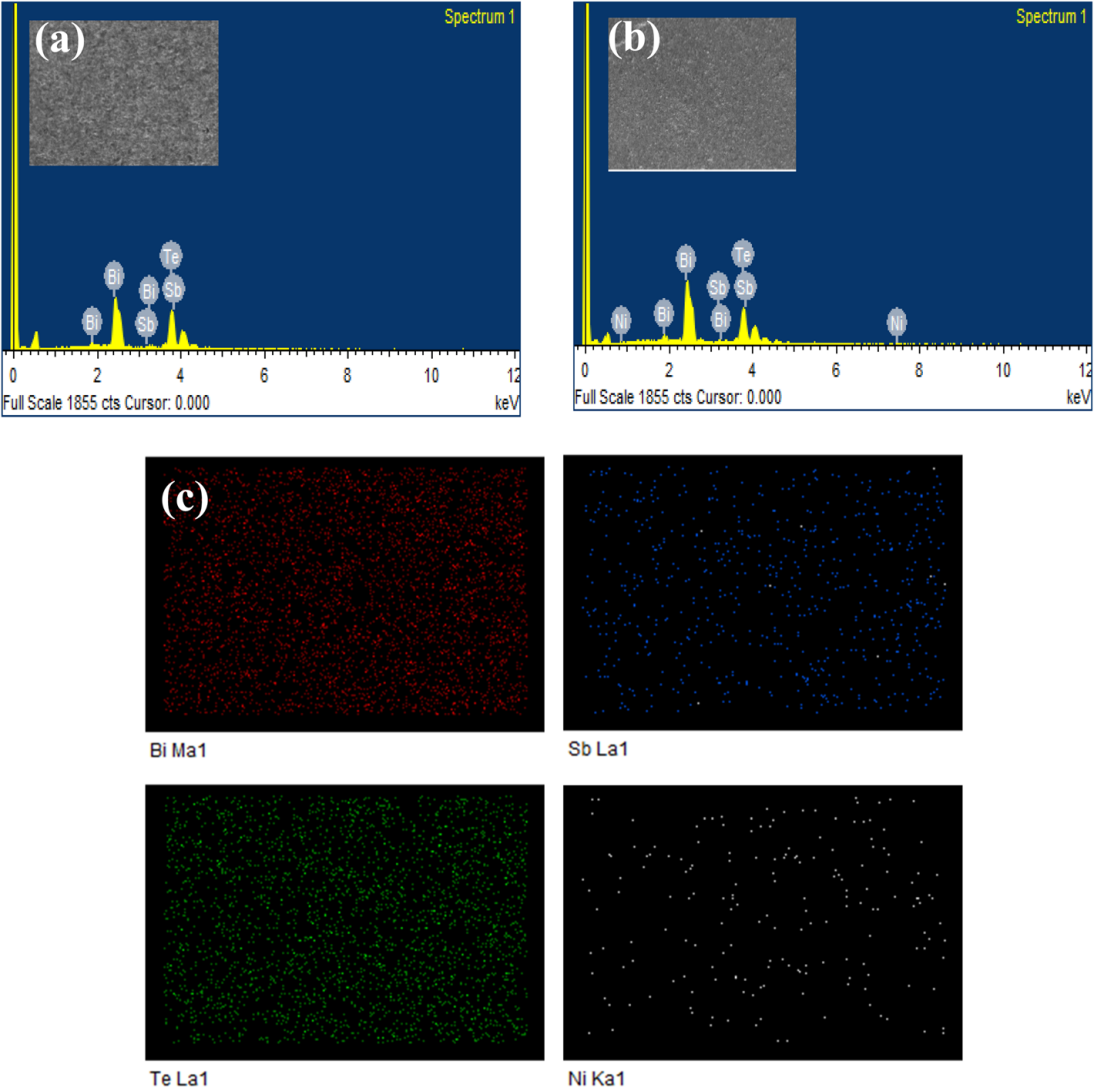
EDX spectra of (a) Bi_1.8_Sb_0.2_Te_3_/0%Ni, (b) Bi_1.8_Sb_0.2_Te_3_/1%Ni, and (c) EDX elemental mapping images of the sample Bi_1.8_Sb_0.2_Te_3_/1%Ni at a selected surface area.

The SEM micrographs of all Bi_1.8_Sb_0.2_Te_3_/*x*%Ni compounds, shown in Fig. 4, reveal the presence of agglomerated particles. The surface of the pristine sample exhibits a hexagonal flake-like structure, while the Ni incorporated samples display small granular surface structures, indicating the presence of nickel. All samples exhibit bulk defects, including porosity. These porous structures are seen more in doped samples compared to the pristine material. Besides, voids in the range of several nanometres are observed. The EDS analysis was employed to identify the elemental presence in the samples. Fig. 5(a) and (b) show the EDS spectra of the Bi_1.8_Sb_0.2_Te_3_ and Bi_1.8_Sb_0.2_Te_3_/1%Ni, respectively, indicating the presence of Bi, Sb, Te, and Ni elements in the samples. The uniform distribution of the elements, especially a dopant, was confirmed by EDS elemental mapping as shown in Fig. 5(c).

### 3.3 X-ray photoelectron spectroscopy

**Fig. 6:**
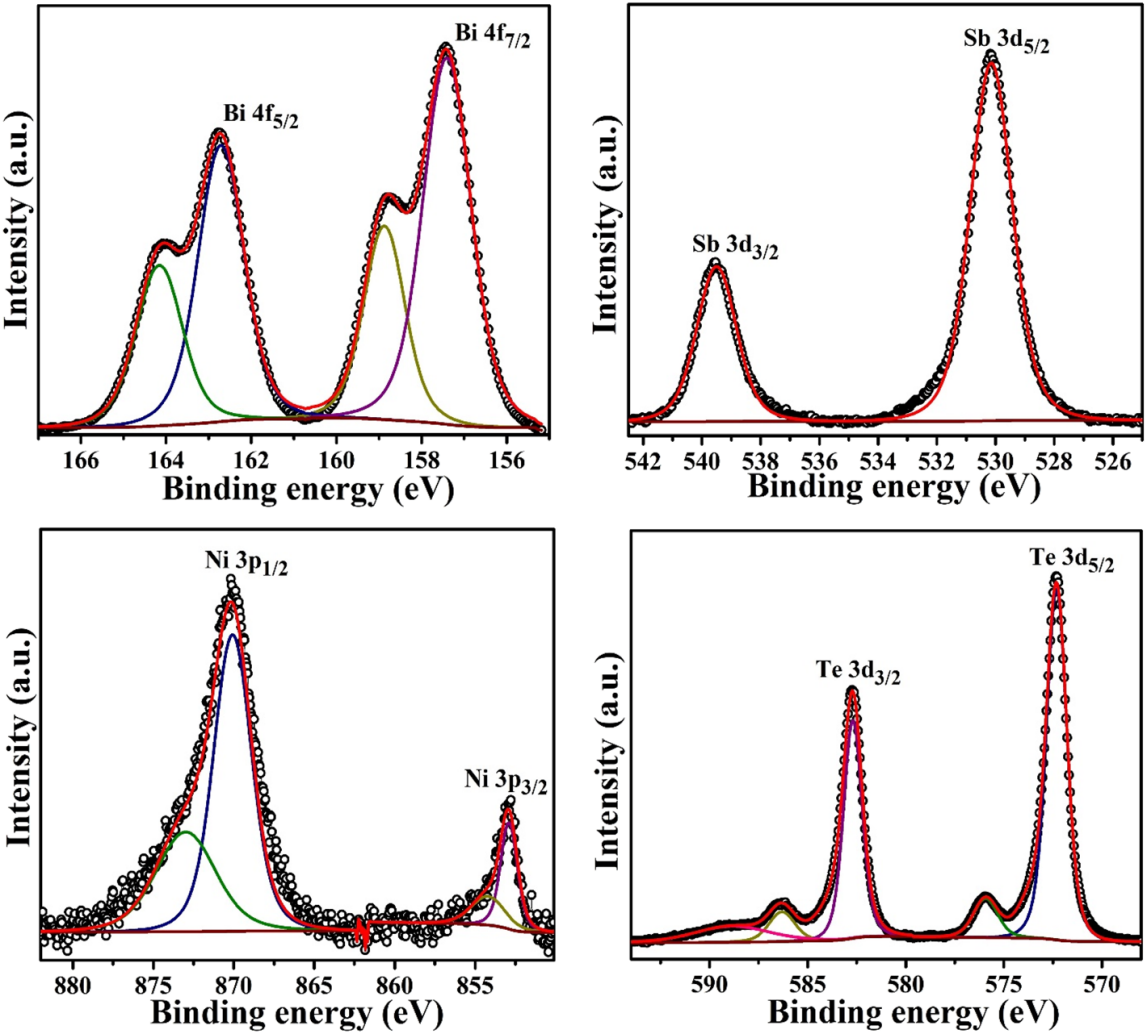
High resolution XPS spectra of Bi_1.8_Sb_0.2_Te_3_/4%Ni sample.

The X-ray photoelectron spectroscopy (XPS) analysis provides definitive evidence for the chemical states and nickel incorporation mechanism in Bi_1.8_Sb_0.2_Te_3_/*x*%Ni composites. Detailed high-resolution XPS spectral analysis of the Bi_1.8_Sb_0.2_Te_3_/4%Ni sample is presented, with the graphs displayed in Fig. 6. The bismuth (Bi 4f) peaks at 157.4 eV (Bi 4f_7/2_) and 162.7 eV (Bi 4f_5/2_) with 5.3 eV spin–orbit splitting perfectly match literature values for Bi^3+^ in Bi_2_Te_3_, confirming preservation of the bismuth chemical environment. Similarly, tellurium (Te 3d) peaks at 572.3 eV (Te 3d_5/2_) and 582.7 eV (Te 3d_3/2_) with 10.4 eV splitting closely align with Te^2−^ in Bi_2_Te_3_ (572.1 eV), indicating structural integrity of the host matrix. The other higher binding energy peaks in the Bi and Te core spectra are attributed to surface oxide formation on the sample.^23^ The antimony (Sb 3d) spectrum shows peaks at 530.14 eV (Sb 3d_5/2_) and 539.5 eV (Sb 3d_3/2_) with 9.4 eV splitting, the observed shift in peak is due to the presence of native Oxygen (O 1s) signals overlapped with the Sb. Most critically, the nickel (Ni 2p) spectrum exhibits a dominant peak at 852.8 eV (Ni 2p_3/2_) and 870.07 eV (Ni 2p_1/2_) with 17.3 eV splitting, which corresponds to the metallic state of nickel (Ni^0^), while the weak higher binding energy components at ∼854.2 eV (Ni 2p_3/2_) and 872.97 eV (Ni 2p_1/2_) indicate minor fractions of nickel that can be attributed to surface oxidation or partial Ni substitution into the Bi/Sb lattice sites.^24^ The Ni 2p_1/2_ peak shows unexpectedly higher intensity compared to the Ni 2p_3/2_ peak, which is opposite to the theoretical 1 : 2 intensity ratio expected from spin–orbit coupling degeneracy. This unusual intensity distribution likely results from overlapping satellite peaks or multiple nickel chemical environments that enhance the higher binding energy region. This XPS analysis conclusively establishes controlled metallic composite formation as the dominant incorporation mechanism, rather than ionic substitution.

### 3.4 Electrical transport properties

**Fig. 7:**
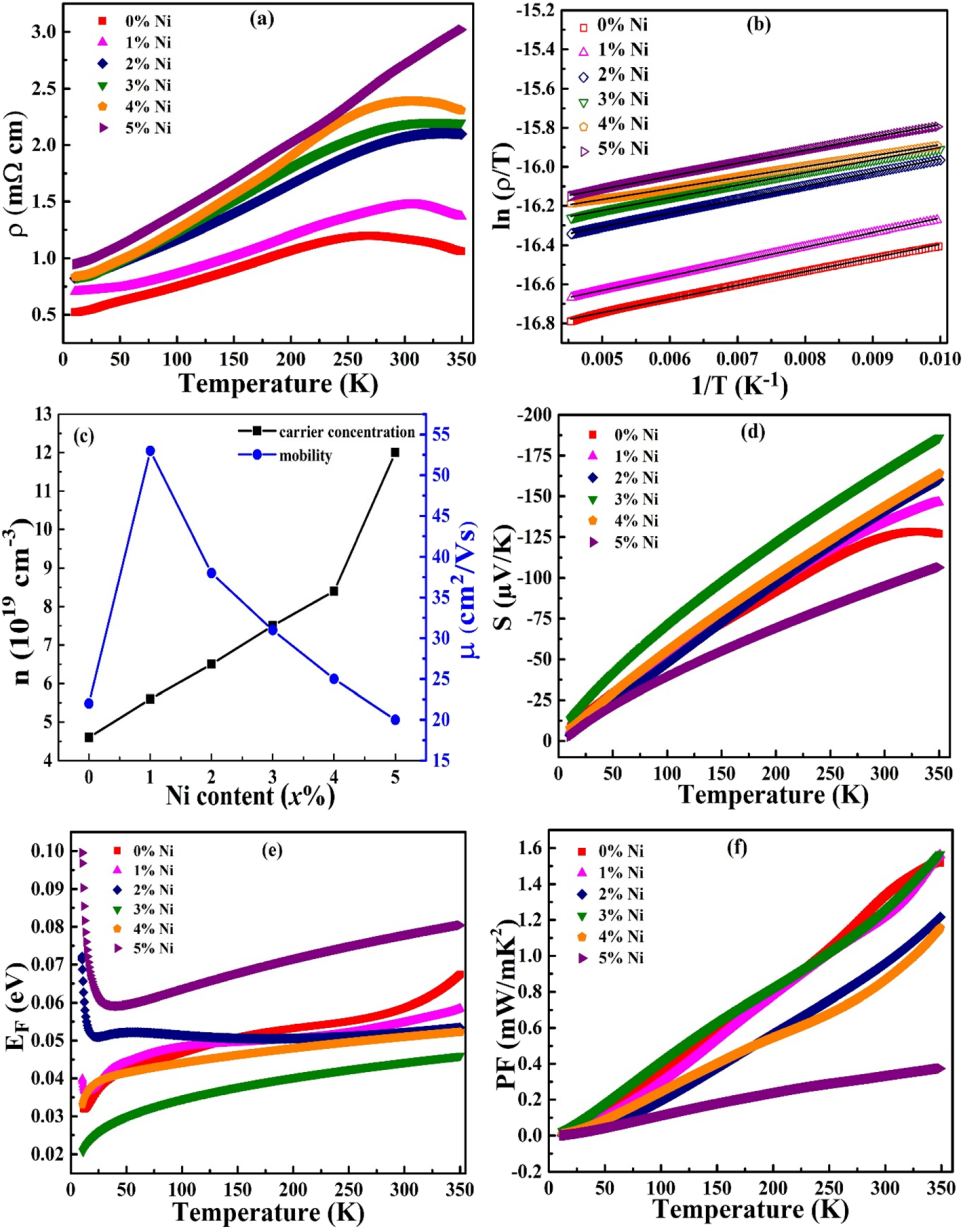
(a) Temperature-dependent electrical resistivity, (b) a fitting of the SPH model, (c) the variations in carrier concentration and mobility with different Ni concentrations, (d) the temperature-dependent Seebeck coefficient, (e) the temperature-dependent Fermi energy and (f) the temperature-dependent power factor of Bi_1.8_Sb_0.2_Te_3_/*x*%Ni (*x* = 0, 1, 2, 3, 4, and 5) samples.

The incorporation of Ni into the Bi_1.8_Sb_0.2_Te_3_ alloy has the potential to enhance carrier concentration. The ferromagnetic characteristics of Ni, when interacting with the host matrix, can alter the band structure by shifting the Fermi level. This shift could increase the density of states and improve the electrical transport properties of the material. As shown in Fig. 7(a), the electrical resistivity of the Bi_1.8_Sb_0.2_Te_3_/*x*%Ni composites exhibits degenerate semiconducting behavior. For the pristine sample, the resistivity increases with temperature up to 250 K. In samples doped with Ni at concentrations of up to 4%, the point of degeneracy shifts to higher temperatures. However, the 5% Ni composite displays completely degenerate semiconducting behavior across the entire investigated temperature range. The degenerate behavior is attributed to a narrow band gap, where the Fermi level is positioned within the conduction band. The transition of degenerate behavior to semiconducting behaviour occurs due to mixed conduction resulting from the thermal excitation of intrinsic charge carriers.^25^ The addition of Ni increases the number of free charge carriers, which stabilizes the degeneracy by shifting the intrinsic excitation temperature to a higher value.^20^

The electrical resistivity of the studied alloys gradually increases with higher doping concentrations. This increase may be attributed to the lattice distortion caused by the introduction of impurities into the complex crystal structure. Lattice distortion can occur due to the Ni ions which might occupy interstitial sites or interlayer positions, which can scatter the charge carriers. This scattering reduces the mobility of the charge carriers, leading to an increase in the electrical resistivity of the samples. To explain the conduction mechanism of the presently investigated system, the small polaron hopping model is employed. The equation for this model is expressed as follows:3
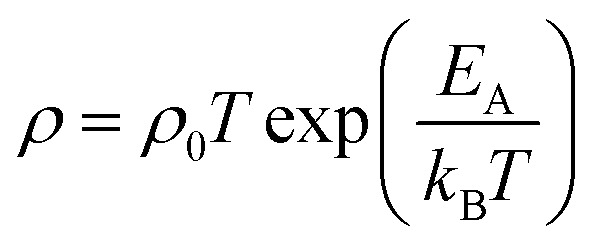
where *ρ* represents the electrical resistivity, *ρ*_0_ denotes the pre-exponential factor, *T* is the temperature, and *E*_A_ stands for the activation energy. The short-range electron-lattice interaction leads to lattice deformation, which creates a potential well and acts as a self-trapped carrier known as a small polaron.^26^ The goodness of fit *R*^2^ values for the SPH model are 0.998, 0.999, 0.999, 0.998, 0.998 and 0.998, showing excellent linear fit for Bi_1.8_Sb_0.2_Te_3_/*x*%Ni composites within 100–220 K temperature range. Here, nickel-induced defects in the lattice create activation barriers that hinder charge carriers from hopping, thereby resulting in low carrier mobility. The energy required to overcome these barriers is referred to as the activation energy.^27^ The activation energy calculated from the slope of Fig. 7(b) is tabulated in Table 3.

**Table 3:** Estimated effective mass, band gap, activation energy, and weighted mobility values for Bi_1.8_Sb_0.2_Te_3_/*x*%Ni (*x* = 0, 1, 2, 3, 4, and 5) samples

Sample	m*/*m*_e_	*E*_g_ (eV)	*μ*_W_ (cm^2^ V^−1^ s^−1^)	*E*_A_ (meV)
*x* = 0	1.16	0.09	27	6.03
*x* = 1	0.97	0.10	50	6.35
*x* = 2	1.12	0.11	45	5.98
*x* = 3	1.46	0.12	54	5.56
*x* = 4	1.37	0.11	40	4.82
*x* = 5	1.16	0.08	25	5.70

Further, parameters related to the room-temperature Hall effect were measured and are summarized in Table 4. The negative sign of the Hall coefficient indicates that the composites display n-type semiconducting behavior, which is attributed to the presence of antisite defects of Bi_Te_ and the anion vacancies. The increase in the antisite defects of Bi and the extra electrons induced by the Ni atoms for the composites enhances the carrier density with the addition of Ni. However, the mobility of the carriers decreases with higher doping levels, as illustrated in Fig. 7(c). Carrier mobility decreases markedly due to enhanced interface scattering at the metal–semiconductor boundaries introduced by dispersed Ni inclusions, where potential barriers impede electron flow and increase electron scattering at the boundaries. Additionally, ionized impurity scattering from charged Ni centers and associated native defects further disrupts carrier trajectories, reducing mean free paths according to the Brooks–Herring formalism.^28^ The combined effects of interfacial and impurity scattering reduces the mobility of the electrons, thereby leading to the observed increase in electrical resistivity.

**Table 4:** Hall effect measurements of Bi_1.8_Sb_0.2_Te_3_/*x*%Ni (*x* = 0, 1, 2, 3, 4, and 5) samples

Sample	Carrier concentration *n* (10^19^ cm^−3^)	Mobility *μ* (cm^2^ V^−1^ s^−1^)	Hall coefficient *R*_H_ (cm^3^ C^−1^)
*x* = 0	4.6	22	−0.13
*x* = 1	5.6	53	−0.11
*x* = 2	6.5	38	−0.09
*x* = 3	7.5	31	−0.08
*x* = 4	8.4	25	−0.07
*x* = 5	12.0	20	−0.05

The temperature-dependent Seebeck coefficient (*S*) across the temperature range of 10 to 350 K is illustrated in Fig. 7(d). A negative Seebeck coefficient suggests that electrons are the dominant charge carriers. Additionally, the Seebeck coefficient increases with temperature, consistent with the behavior of degenerate semiconductors. A transition from degenerate to non-degenerate semiconducting behavior is observed at high temperatures in the pristine sample. However, this transition is not evident in the Ni composite samples because, it shifts to higher temperatures beyond the experimental limits. Up to 3% Ni doping, the Seebeck coefficient gradually increases, but it decreases with further increases in doping concentration. The highest Seebeck coefficient is 185 μV K^−1^ at a Ni concentration of 3% at 350 K. The pristine sample exhibited an *S* value of 125 μV K^−1^ at room temperature, consistent with the value reported in the literature.^8^ This finding can be attributed to the optimal carrier concentration and mobility observed in the sample, as illustrated in Fig. 7(c). The Seebeck coefficient primarily depends on the carrier concentration, a relationship that is described by the Mott relation:4
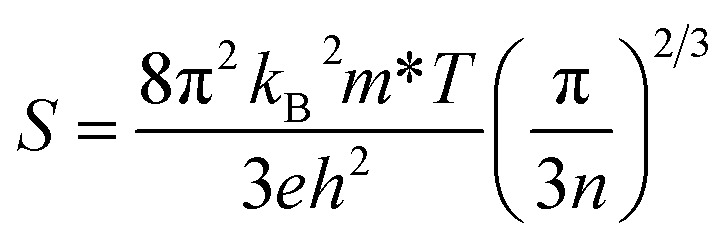
where m* is the effective mass and *n* is the carrier concentration.^29^ The effective masses of the samples, calculated using the eqn (4), are listed in Table 3. As expected, the Bi_1.8_Sb_0.2_Te_3_/3%Ni sample shows the highest effective mass. Temperature-dependent Fermi level analysis of Bi_1.8_Sb_0.2_Te_3_/*x*%Ni composites (shown in Fig. 7(e)) reveals that increasing Ni concentration shifts the Fermi level deeper into the conduction band, stabilizing degenerate semiconductor behaviour at elevated temperatures. These Fermi energy is estimated by using the relation5
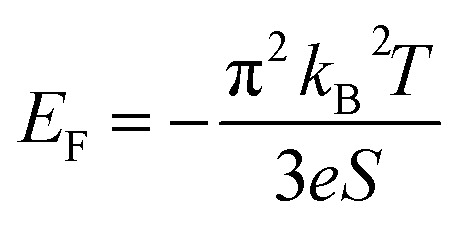
Within the Single Parabolic Band (SPB) framework, this shift correlates with an enhanced density of states (DOS), expressed as *g*(*E*) ∝ *N*_v_ (m*)^3/2^, where both valley degeneracy *N*_v_ and effective mass m* increase due to band convergence. The effective mass enhancement from 0.97 to 1.46 at 3% Ni doping reflects this DOS modification, contributing to improved Seebeck coefficient *via* the Mott relation, where higher effective mass increases the energy derivative of carrier concentration.^30,31^ Ni addition perturbs the band structure by introducing impurity states and additional conduction or valence band valleys, with the resultant Fermi level shift consistent with increased carrier concentration observed in Hall measurements, confirming stabilized degenerate behavior at higher Ni contents.^31^ Additionally, the weighted mobility is defined as carrier mobility weighted by the density of states and it is calculated using the effective mass, which is given by the equation.6
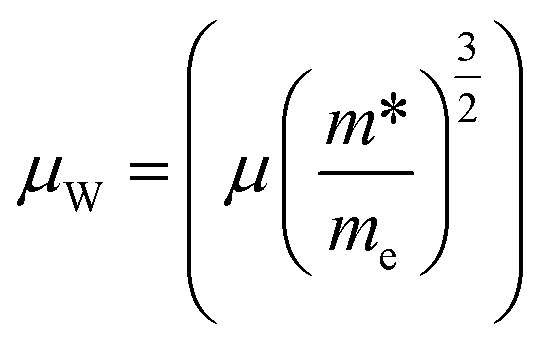
where *μ*_W_ is the weighted mobility and *m*_e_ is the mass of an electron. The calculated *μ*_W_ values at room temperature are presented in Table 3. Materials with parabolic energy bands typically show no change in weighted mobility with varying doping concentrations. In contrast, materials with non-parabolic energy bands demonstrate changes in weighted mobility as the doping concentration varies, indicating a more complex band structure and associated scattering mechanisms.^32^ The weighted mobilities in the doped samples are higher than those in the pristine sample, which aligns with the variations in the Seebeck coefficient observed at different doping levels. Additionally, the thermal energy bandgap (*E*_g_) near the Fermi level can be quantitatively determined through direct calculations based on the experimental values of the Seebeck coefficient. This relationship is expressed by the following equation:7
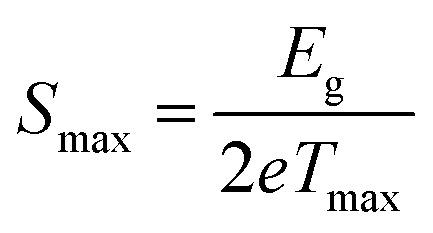
where *T*_max_ is the temperature at which the Seebeck coefficient is maximum, denoted by *S*_max_.^33^ The prepared Bi_1.8_Sb_0.2_Te_3_/*x*%Ni (*x* = 0, 1, 2, 3, 4, and 5) samples exhibit a narrow bandgap that ranges from 0.08 to 0.12 eV. The bandgap value of the pristine sample is slightly lower than that of the binary bismuth telluride material reported in the literature.^34,35^

The temperature-dependent power factor of all studied samples is presented in Fig. 7(f). It was calculated using the Seebeck coefficient values and resistivity, according to the equation PF = *S*^2^/*ρ*. The power factor increases with temperature, reaching a maximum of 1.56 mW mK^−2^ in samples with *x* = 1% and *x* = 3% Ni, which is comparable to the value of 1.51 mW mK^−2^ for the pristine sample. For the remaining samples, however, the power factor decreases, with the lowest value recorded at 0.38 mW mK^−2^ at 350 K for the *x* = 5% Ni sample. This low value is attributed to the high resistivity and low Seebeck coefficient of the sample.

### 3.5 Thermal transport properties

**Fig. 8:**
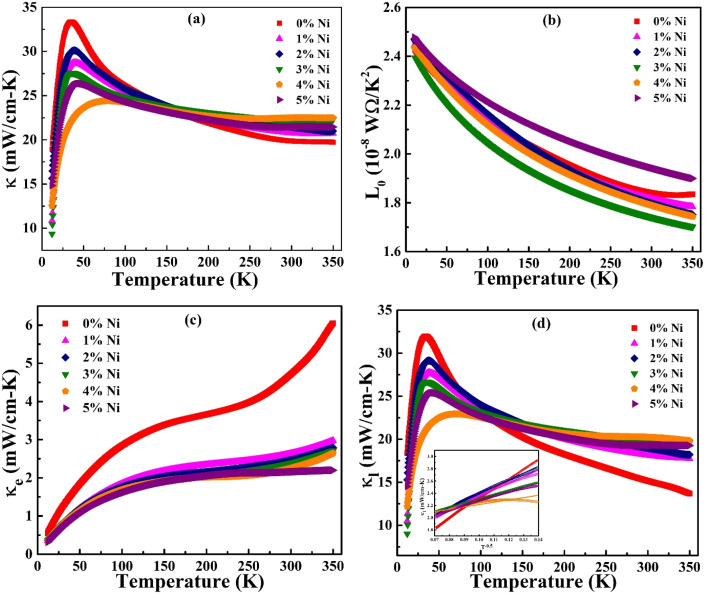
Temperature-dependent (a) total thermal conductivity, (b) Lorenz number (*L*_0_), (c) electronic thermal conductivity, and (d) lattice thermal conductivity of Bi_1.8_Sb_0.2_Te_3_/*x*%Ni (*x* = 0, 1, 2, 3, 4, and 5) samples.

The BiSbTe alloy exhibits inherently low thermal conductivity, attributed to strong anharmonic lattice vibrations and a complex crystal structure that effectively scatters phonons. The introduction of transition metal Ni may further reduce thermal conductivity by inducing point defects and lattice distortions within the material. The total thermal conductivity (*κ*) of the prepared samples, measured over a temperature range of 10 to 350 K, is illustrated in Fig. 8(a). A sharp increase in thermal conductivity is observed in the low-temperature regime, with a peak at approximately 40 K. Furthermore, it decreases gradually at higher temperatures. This behavior is commonly seen in crystalline materials. At low temperatures, the thermal energy is insufficient to surpass the first Brillouin zone boundary, which suppresses scattering processes due to momentum redistribution. As a result, thermal resistance is low, and long-wavelength acoustic phonons become more prevalent, leading to high thermal conductivity. At higher temperatures, a significant number of high-energy, short-wavelength phonons are thermally excited. This process leads to predominant phonon–phonon Umklapp scattering, which reduces the mean free path of phonons and, consequently, diminishes thermal conductivity.^36,37^ At near room temperature, the doped samples show a slightly higher thermal conductivity than the pristine sample, which may be attributed to an increase in the mean free path and relaxation processes. The thermal conductivity of the pristine sample at room temperature is approximately 19 mW cm^−1^ K^−1^, which is comparable to the earlier reports.^11^

In metals and degenerate semiconductors, thermal conductivity consists of contributions from both charge carriers and the lattice, expressed as *κ* = *κ*_e_ + *κ*_l_. The electronic thermal conductivity (*κ*_e_) can be calculated using the Wiedemann–Franz law, given by the equation8
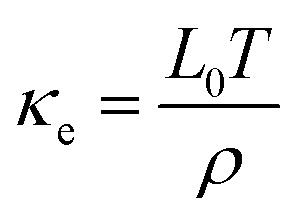
where *ρ* is the electrical resistivity and *L*_0_ is the Lorenz number. The prepared samples display degenerate semiconducting behavior that is significantly influenced by scattering mechanisms. As a result, the value of *L*_0_ depends on the temperature. Kim *et al.* derived an expression for the Lorenz number, which is calculated using the Seebeck coefficient values given by:^38^9
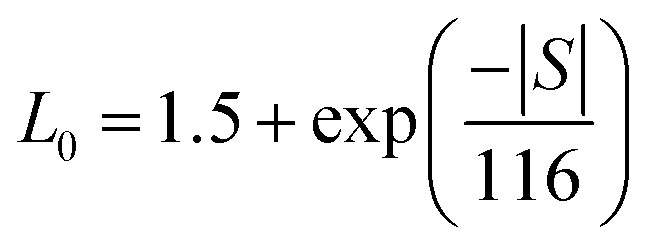
By substituting the values of the Seebeck coefficient (*S*), the Lorenz number is calculated as shown in Fig. 8(b). The temperature-dependent values of *L*_0_ for all the studied compositions are found to be close to the degenerate limit (2.443 × 10^−8^ W Ω K^−2^). The Lorenz number decreases as the Seebeck coefficient increases with temperature, which can be attributed to charge scattering mechanisms. These calculated *L*_0_ values are then used to determine the electronic thermal conductivity using eqn (9). As evident from the plot shown in Fig. 8(c), the electronic thermal conductivity increases with temperature, and the Bi_1.8_Sb_0.2_Te_3_ sample demonstrates the highest *κ*_e_ value of about 6 mW cm^−1^ K^−1^ at 350 K. However, the introduction of Ni into the matrix decreases the electronic thermal conductivity. This reduction may result from an increase in interfacial Ni sites, which enhances the scattering of the majority charge carriers, thereby reducing the mobility of the electrons.

The lattice thermal conductivity (*κ*_l_) is determined by subtracting the electronic thermal conductivity from the total thermal conductivity, as illustrated in Fig. 8(d). The temperature dependence of lattice thermal conductivity is influenced by various scattering mechanisms. At low temperatures, where *T* ≪ *Θ*_D_ (Debye temperature), the normal process leads to an increase in *κ*_l_, with the volume specific heat being directly proportional to *T*^3^. Above the Debye temperature, lattice thermal conductivity typically follows a *T*^−1^ dependence; however, in the present study *κ*_l_ varies as *T*^−0.5^ (inset, Fig. 7d), indicating the presence of strong point-defect scattering aligning with the Callaway model framework. The *x* = 4% Ni sample exhibits the lowest *R*^2^ value in the *T*^−0.5^ fit, signifying reduced point-defect scattering compared to other Ni-doped compositions. The pristine sample demonstrates the highest *R*^2^ fit compared to Ni composite samples, suggesting that the incorporation of Ni reduces point-defect scattering within the Bi_1.8_Sb_0.2_Te_3_ matrix.^39^ The observed increase in lattice thermal conductivity (*κ*_l_) upon Ni addition in Bi_1.8_Sb_0.2_Te_3_ is primarily attributed to the metallic nature of Ni within the composite, as confirmed by X-ray photoelectron spectroscopy (XPS). These metallic Ni inclusions act as highly efficient heat conduction pathways, enhancing overall thermal transport across the material. While phonon scattering at the interfaces remains present, Ni incorporation predominantly intensifies electron–electron scattering rather than phonon scattering. Moreover, since Ni exists mainly as metallic inclusions rather than substitutional dopants, the typical mass-difference phonon scattering is minimized, allowing the high intrinsic thermal conductivity of metallic Ni to dominate the lattice thermal conductivity behavior. The lowest lattice thermal conductivity observed is approximately 13.6 mW cm^−1^ K^−1^ for the pristine sample at 350 K. Furthermore, the total thermal conductivity of all studied samples is mainly due to lattice contributions rather than electronic contributions.

### 3.6 Quality factor (*B*) and figure of merit (*ZT*)

**Fig. 9:**
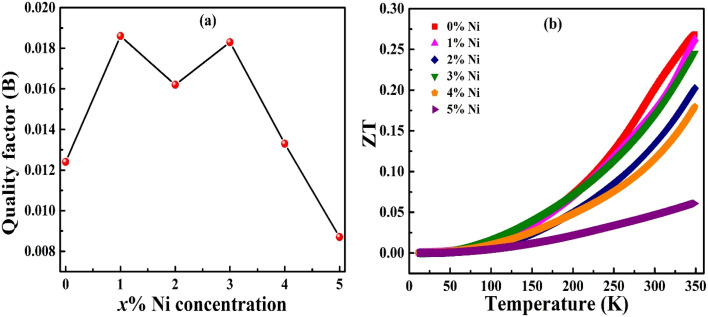
(a) Thermoelectric quality factor at room temperature and (b) figure of merit (*ZT*) as a function of temperature of Bi_1.8_Sb_0.2_Te_3_/*x*%Ni (*x* = 0, 1, 2, 3, 4, and 5) samples.

The thermoelectric quality factor (*B*) is a key metric for assessing the intrinsic potential of a material. This parameter highlights the synergistic effect of increased weighted mobility *μ*_W_ and reduced lattice thermal conductivity in enhancing the thermoelectric performance of the material. The room temperature quality factor is calculated by using equation^40^10
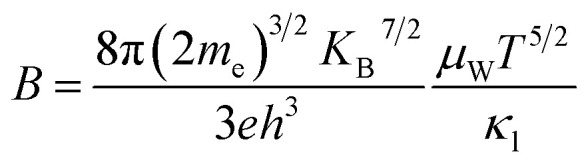
In the present Bi_1.8_Sb_0.2_Te_3_/*x*%Ni series, the maximum *B* value of approximately 0.0185 was obtained for the 1% Ni sample as shown int the Fig. 9(a), reflecting its optimal combination of moderately enhanced effective mass, retained high mobility, and relatively low lattice thermal conductivity. This peak underscores the importance of fine-tuning dopant concentration to achieve the best trade-off between electronic transport and phonon suppression. The dimensionless figure of merit (*ZT*) is calculated using the measured electrical and thermal transport parameters with the formula *ZT* = *S*^2^*T*/*ρκ*_l_. Fig. 9(b) shows the estimated figure of merit as a function of temperature for the Bi_1.8_Sb_0.2_Te_3_/*x*%Ni (*x* = 0, 1, 2, 3, 4, and 5) samples, and the figure of merit is found to increase with increasing temperature. However, no significant enhancement in the power factor is observed compared to the pristine sample. Additionally, the increase in lattice thermal conductivity leads to a decrease in *ZT* as the Ni concentration increases. The maximum *ZT* value of 0.27 is recorded at 350 K for the pristine sample. Among the doped samples, the highest *ZT* observed is 0.26 for the *x* = 1% Ni sample, which is comparable to the *ZT* of the pristine sample. Table 5 presents the comparison of highest figure of merit obtained for various Ni-doped Bismuth telluride based system reported in the literature.

**Table 5:** Comparison of highest *ZT* values of Ni incorporated Bi–Sb–Te alloys reported in the literature

Sl no.	Compound	Methodology	*ZT*	*T* (K)	Ref.
1	Bi_0.5_Sb_1.5_Te_3_/1wt%Ni	Resistance pressing sintering	1.07	330	17
2	Bi_0.5_Sb_1.46_Ni_0.04_Te_3_	Spark plasma sintering (SPS)	1.38	433	18
3	Bi_2_Te_2.7_Se_0.3_/0.3%Ni	Solvothermal and SPS	1.1	360	41
4	Bi_0.5_Ni_0.04_Sb_1.5_Te_3_	SPS	1.4	373	16
5	Bi_0.5_Sb_1.5_Te_3_/1wt%Ni	Hot pressing method	0.55	293	42
6	Bi_0.5_Sb_1.5_Te_3_/1%Ni	Solid state reaction	0.26	350	Present work

## 4. Conclusion

In summary, the solid-state reaction technique is employed to synthesise the Bi_1.8_Sb_0.2_Te_3_/*x*%Ni (*x* = 0, 1, 2, 3, 4, and 5) samples. The powder XRD profile confirms the presence of a rhombohedral crystal structure, and no secondary phases are observed in the doped samples. The thermoelectric properties are investigated at low temperatures, ranging from 10 to 350 K. A smooth transition from degenerate to nondegenerate semiconducting behavior is observed in electrical resistivity measurements, indicating a mixed conduction mechanism at higher temperatures. The negative Seebeck coefficient indicates that electrons are the primary charge carriers responsible for thermoelectric conduction in this series of samples. The lattice thermal conductivity reveals the significance of Umklapp scattering, along with the reduced scattering effects noticed for the addition of Ni samples. The highest power factor, approximately of 1.56 mW mK^−2^, is observed in samples with *x* = 0 and 1% Ni at 350 K. The highest *ZT* was found to be 0.27 for the pristine sample at 350 K. Among all Ni composite samples 1% Ni got the highest *ZT* of 0.26 and quality factor of 0.018. The work gives the comprehensive insights into the impact of Ni on the thermoelectric properties of the n-type Bi_1.8_Sb_0.2_Te_3_ material at low temperatures.

## Conflicts of interest

There are no conflicts to declare.

## Data Availability

The data cannot be made publicly available upon publication as they are not available in a standard format that is sufficiently accessible by other researchers. The data that support the outcomes of this study will be shared upon reasonable request from the authors.
